# Supplementary material for: A d-2-hydroxyglutarate biosensor based on specific transcriptional regulator DhdR
Source: Nat Commun. 2021 Dec 7;12:7108. doi: 10.1038/s41467-021-27357-7 (PMC8651671; doi:10.1038/s41467-021-27357-7)
Supplement: Supplementary file 1 — Supplementary Information [file 41467_2021_27357_MOESM1_ESM.pdf]

Supplementary Information

A D-2-hydroxyglutarate biosensor based on specific transcriptional regulator DhdR

Dan Xiao[1,5], Wen Zhang[2,5], Xiaoting Guo[3], Yidong Liu[1], Chunxia Hu[1], Shiting Guo[1], Zhaoqi Kang[1], Xianzhi Xu[1], Cuiqing Ma[1], Chao Gao[1,*], Ping Xu[4,*]

[1]*State Key Laboratory of Microbial Technology, Shandong University, Qingdao, People's Republic of China*

[2]*Institute of Medical Sciences, The Second Hospital, Cheeloo College of Medicine, Shandong University, Jinan, People's Republic of China*

[3]*Eye Hospital of Shandong First Medical University, Jinan, People's Republic of China*

[4]*State Key Laboratory of Microbial Metabolism, Joint International Research Laboratory of Metabolic & Developmental Sciences, and School of Life Sciences & Biotechnology, Shanghai Jiao Tong University, Shanghai, People's Republic of China*

[5]*These authors contributed equally: Dan Xiao, Wen Zhang.*

***Corresponding authors:**

Mailing address for C. Gao: State Key Laboratory of Microbial Technology, Shandong University, Qingdao 266237, People's Republic of China, Tel/Fax: +86-532-58631561, E-mail: jieerbu@sdu.edu.cn.

Mailing address for P. Xu: State Key Laboratory of Microbial Metabolism, and School of Life Sciences & Biotechnology, Shanghai Jiao Tong University, Shanghai 200240, People's Republic of China, Tel/Fax: +86-21-34206723, E-mail: pingxu@sjtu.edu.cn.

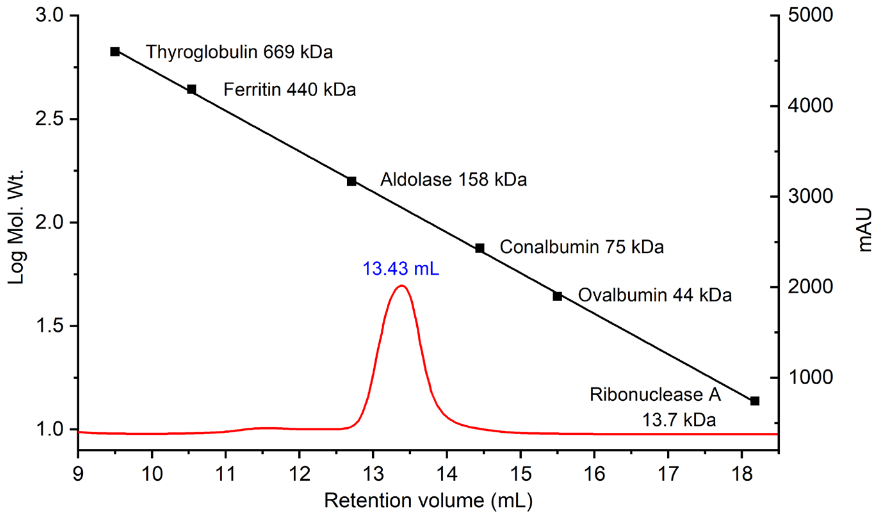

Supplementary Figure 1. Determination of the molecular weight of D2HGDH in *A. denitrificans* NBRC 15125 by size exclusion chromatography. A calibration curve (black line) was obtained using protein molecular mass standards. The molecular weight of D2HGDH was calculated according to the retention volume (13.43 mL) in the chromatogram (red curve). Source data are provided as a Source Data file.

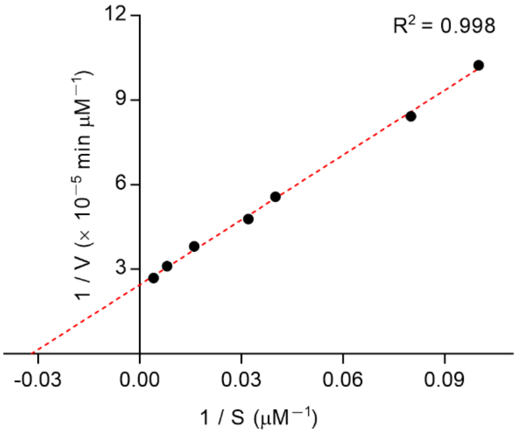

Supplementary Figure 2. Lineweaver–Burk plot for the purified D2HGDH toward D-2-HG. The reaction mixtures contained 0.01 mg mL^{-1} purified D2HGDH, 0.2 mM PMS, 0.05 mM DCPIP, and variable concentrations of D-2-HG in 50 mM Tris-HCl (pH 7.4). The activity of D2HGDH was assayed at 30 ºC by determining the reduction of DCPIP spectrophotometrically at 600 nm. Data shown are mean ± s.d. (n = 3 independent experiments). Source data are provided as a Source Data file.

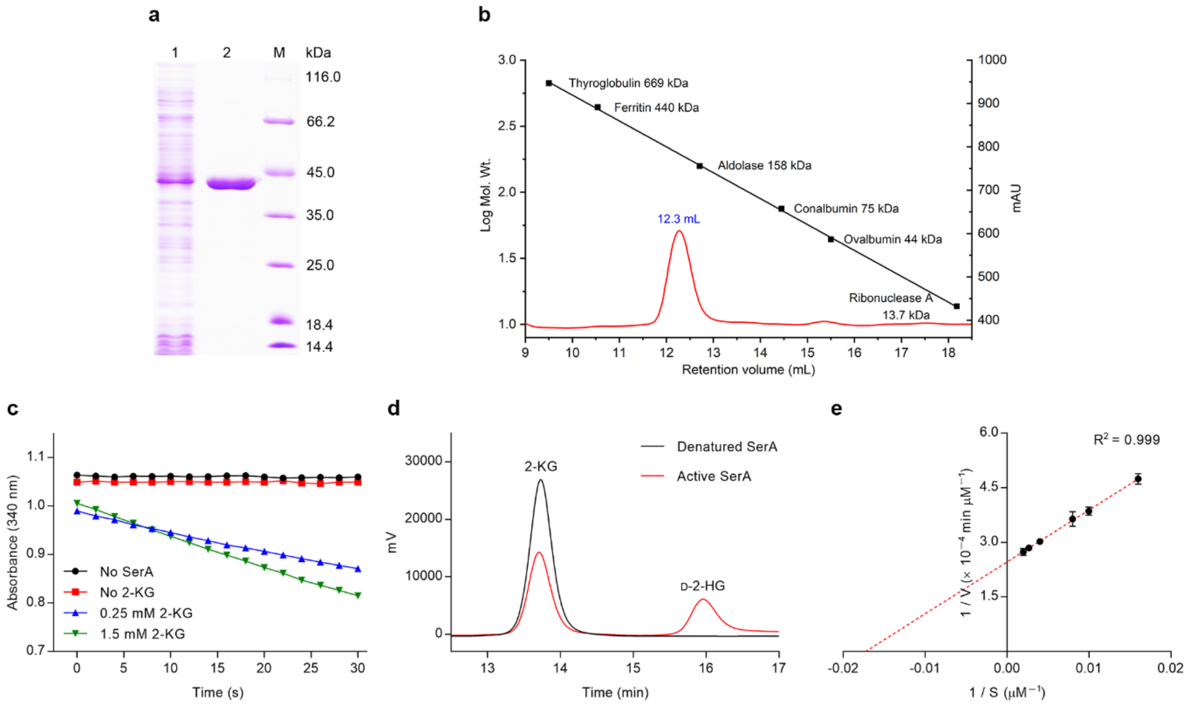

Supplementary Figure 3. Purification and characterization of SerA in *A. denitrificans* NBRC 15125. a, SDS-PAGE analysis of the purified SerA. Lane 1, crude extract of *E. coli* BL21(DE3) harboring pETDuet-*serA*; lane 2, purified SerA using a HisTrap column; lane M, molecular weight markers. **b**, Determination of the molecular weight of SerA by size exclusion chromatography. Black line, standard curve for protein molecular mass standards; red curve, chromatogram of purified SerA. **c**, Activity of SerA toward 2-KG reduction. **d**, HPLC analysis of the product of SerA-catalyzed 2-KG reduction. The reaction mixtures containing 2-KG (50 mM), NADH (40 mM), and active or denatured 2-KG (0.34 mg mL^{-1}) in 50 mM Tris-HCl (pH 7.4) were incubated at 37 ºC overnight. Black line, the reaction with denatured SerA; red line, the reaction with active SerA. **e**, The Lineweaver–Burk plot for the purified SerA toward 2-KG. The reaction mixtures contained 0.05 mg mL^{-1} purified SerA, 0.2 mM NADH, and variable concentrations of 2-KG in 50 mM Tris-HCl (pH 7.4). The activity of SerA was assayed at 30 ºC by measuring the oxidation of NADH spectrophotometrically at 340 nm. Data shown are mean ± s.d. (n = 3 independent experiments). Source data are provided as a Source Data file.

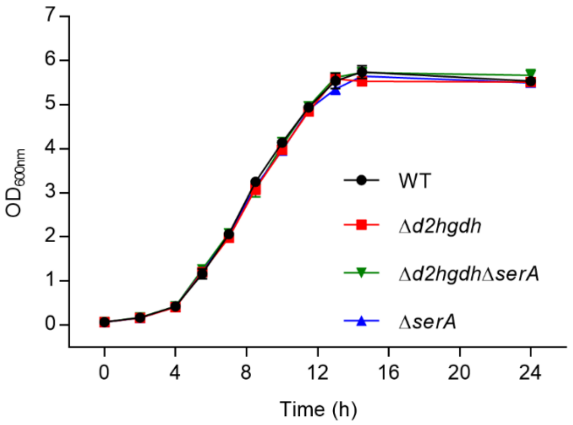

Supplementary Figure 4. Growth of *A. denitrificans* NBRC 15125 and its derivatives cultured in LB medium. Data shown are mean ± s.d. (*n* = 3 independent experiments). Source data are provided as a Source Data file.

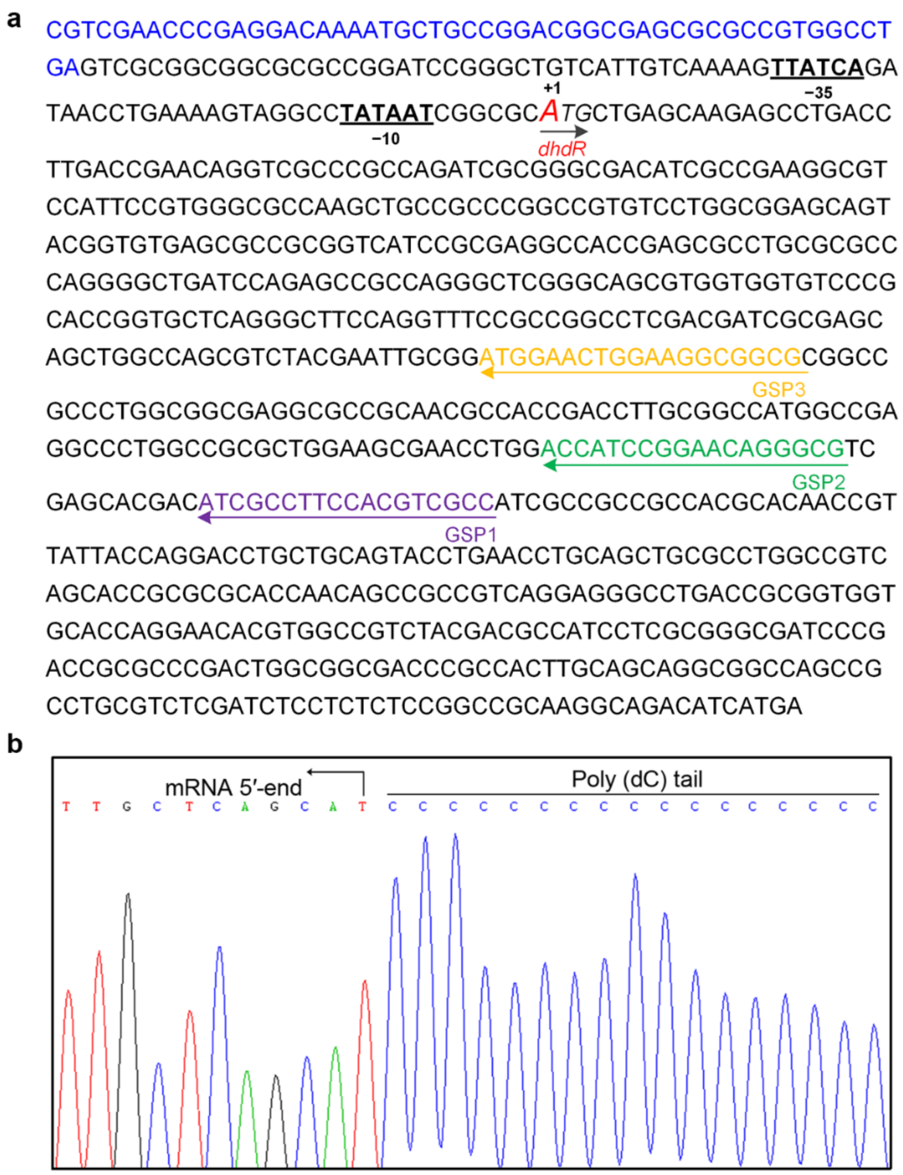

Supplementary Figure 5. Determination of the transcriptional start site (TSS) of *dhdR* by rapid amplification of cDNA ends (RACE). a, Map of the intergenic region upstream of *dhdR-d2hgdh* operon and *dhdR*. TSS is shown in enlarged red letter and marked by +1. The putative −35 and −10 regions are shown in bold and underlined. Amplicon locations are indicated by primers GSP1, GSP2, and GSP3. **b**, Chromatogram displays the partial sequences of the 5′ RACE products, which are the complementary sequences of the nucleotide sequence shown in **a**. The TSS of *dhdR* is indicated by bent arrow.

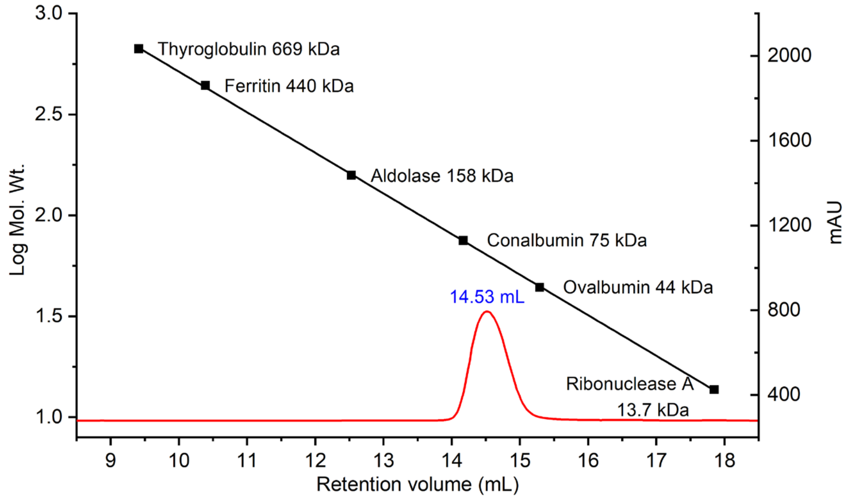

Supplementary Figure 6. Determination of the molecular weight of DhdR by size exclusion chromatography. Black line, standard curve for protein molecular mass standards; red curve, chromatogram of purified DhdR. Source data are provided as a Source Data file.

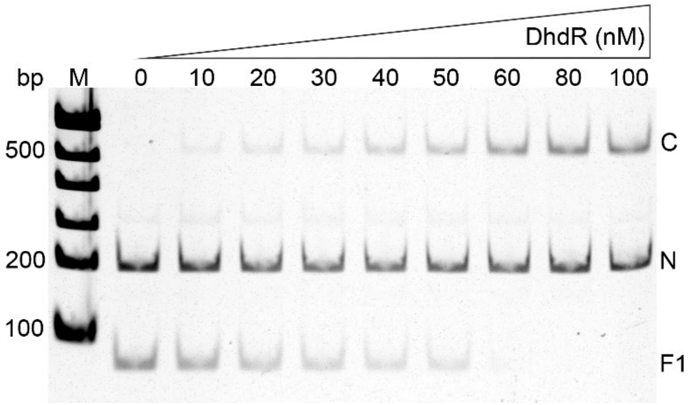

Supplementary Figure 7. EMSAs with 81-bp fragment (F1) upstream of *dhdR* (10 nM) and purified DhdR (0, 10, 20, 30, 40, 50, 60, 80, and 100 nM). A 200-bp internal fragment of *dhdR* (10 nM) was used as a negative control. The position of free F1, complex of DhdR-F1 (C), and negative control (N) are indicated. Lane M, DNA ladder marker. Source data are provided as a Source Data file.

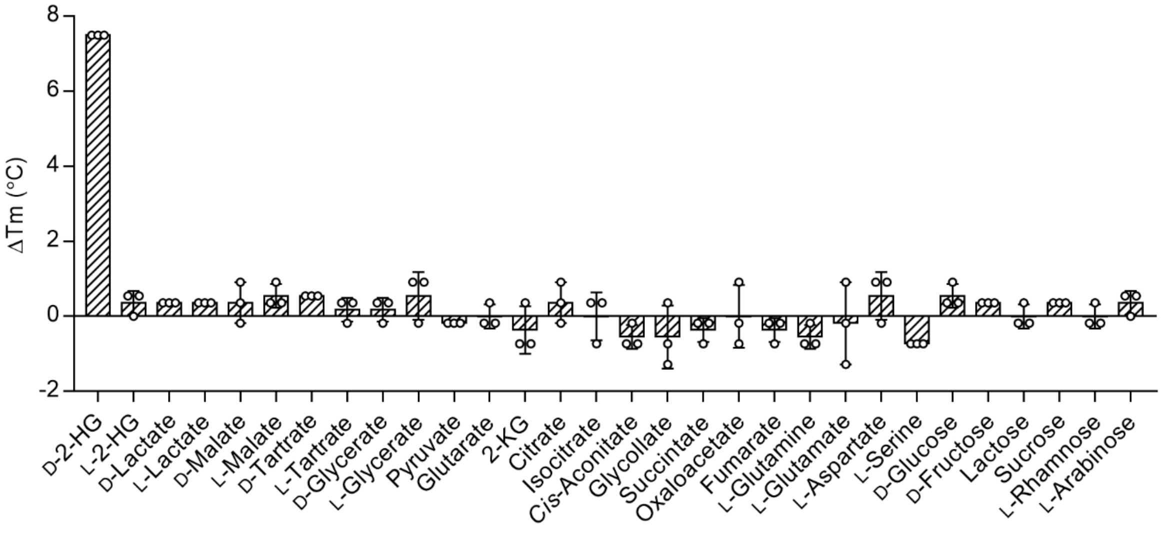

Supplementary Figure 8. Identification of the ligand of DhdR by fluorescence-based thermal shift (FTS) assays. The ΔTm values indicate the changes in Tm values compared to control (DhdR without ligand). The concentration of tested compounds was 100 μM. Data shown are mean ± standard deviations (s.d.) (*n* = 3 independent experiments). Source data are provided as a Source Data file.

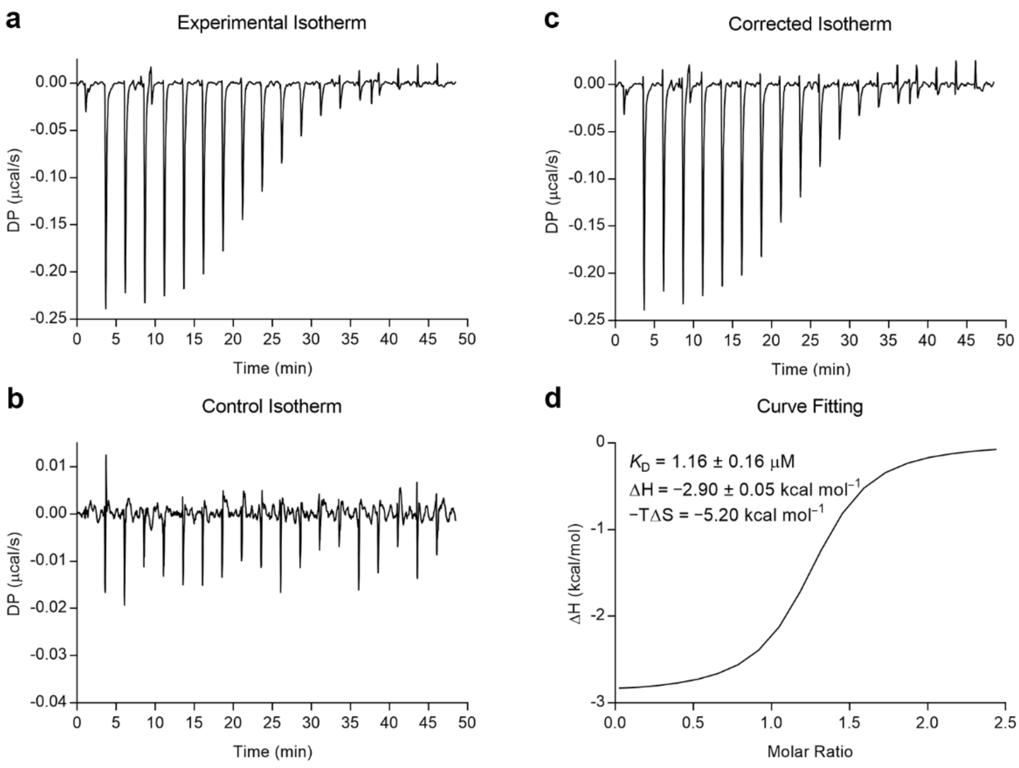

Supplementary Figure 9. Isothermal titration calorimetry (ITC) of D-2-HG binding to DhdR. a, Experiment isotherm. 500 µM D-2-HG was titrated into 40 µM DhdR with 19 injections. **b**, Control isotherm. 500 µM D-2-HG was titrated into reaction buffer. **c**, Corrected isotherm. The net heat of dilutions was corrected by subtracting the heat of the control point-to-point. **d**, Curve fitting. A one site fitting model was used for curve fitting by using MicroCal PEAQ-ITC analysis software. Source data are provided as a Source Data file.

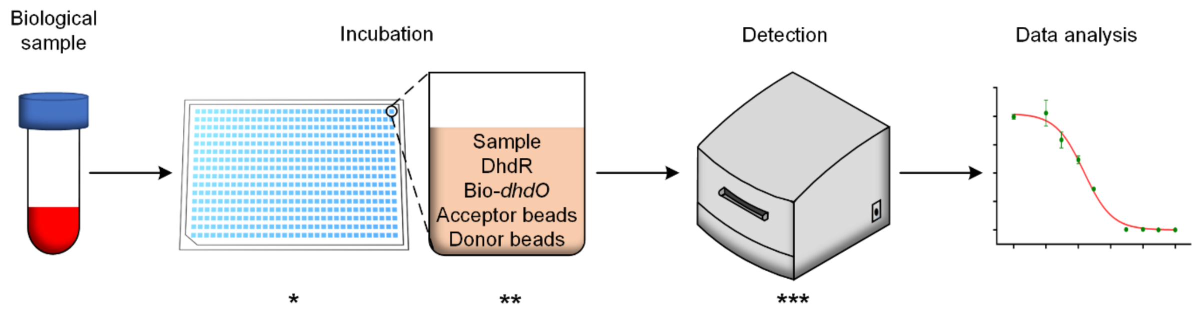

Supplementary Figure 10. Flow chart for the process of determining D-2-HG in biological sample via the developed biosensor. *, 384-well plates; **, the components contained in each well; ***, multimode plate reader.

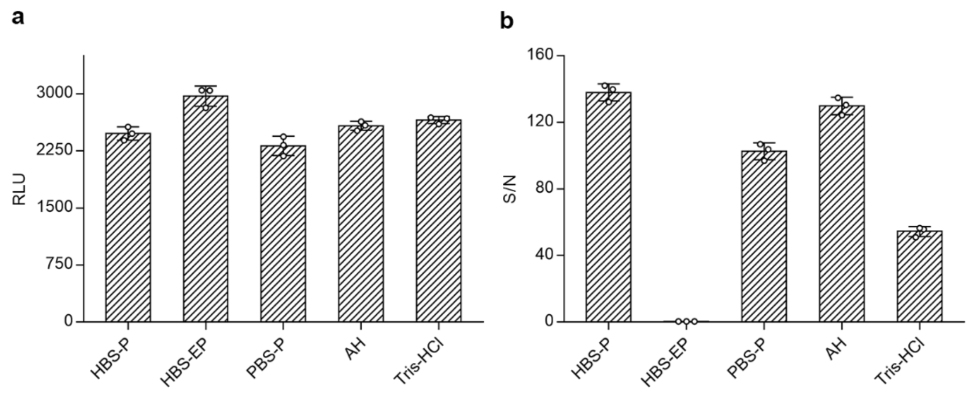

Supplementary Figure 11. Selection of the optimal buffer for biosensor based on background signal and the ratio of signal-to-noise (S/N). **a**, The background signal of the biosensor in different buffers. The background signals were measured in 25 μL detecting solution containing 5 μL donor beads (20 μg mL^{-1}), 5 μL acceptor beads (20 μg mL^{-1}) and 15 μL indicated buffer. **b**, The S/N of the biosensor in different buffers. The detecting solution (25 μL) contained 5 μL of each component: DhdR (0.3 nM), Bio-*dhdO* (1 nM), donor beads (20 μg mL^{-1}), acceptor beads (20 μg mL^{-1}) and corresponding buffer. Data shown are mean ± s.d. (n = 3 independent experiments). Source data are provided as a Source Data file.

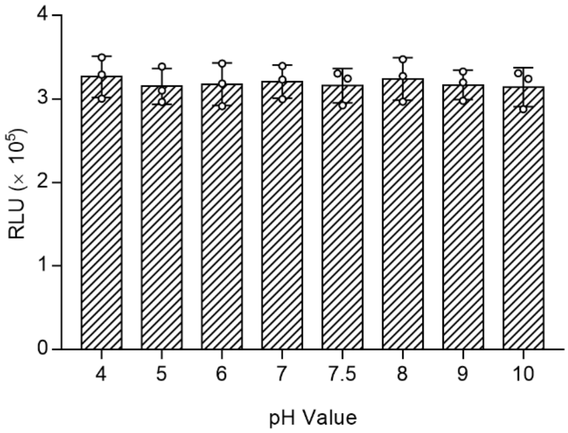

Supplementary Figure 12. Evaluation of the effect of sample pH on D-2-HG detection.

The detecting solution (25 μL) contained 5 μL of each component: DhdR (0.3 nM), Bio-*dhdO* (1 nM), donor beads (20 μg mL^{-1}), acceptor beads (20 μg mL^{-1}), and HBS-P buffer with different pH values. Data shown are mean ± s.d. (*n* = 3 independent experiments). Source data are provided as a Source Data file.

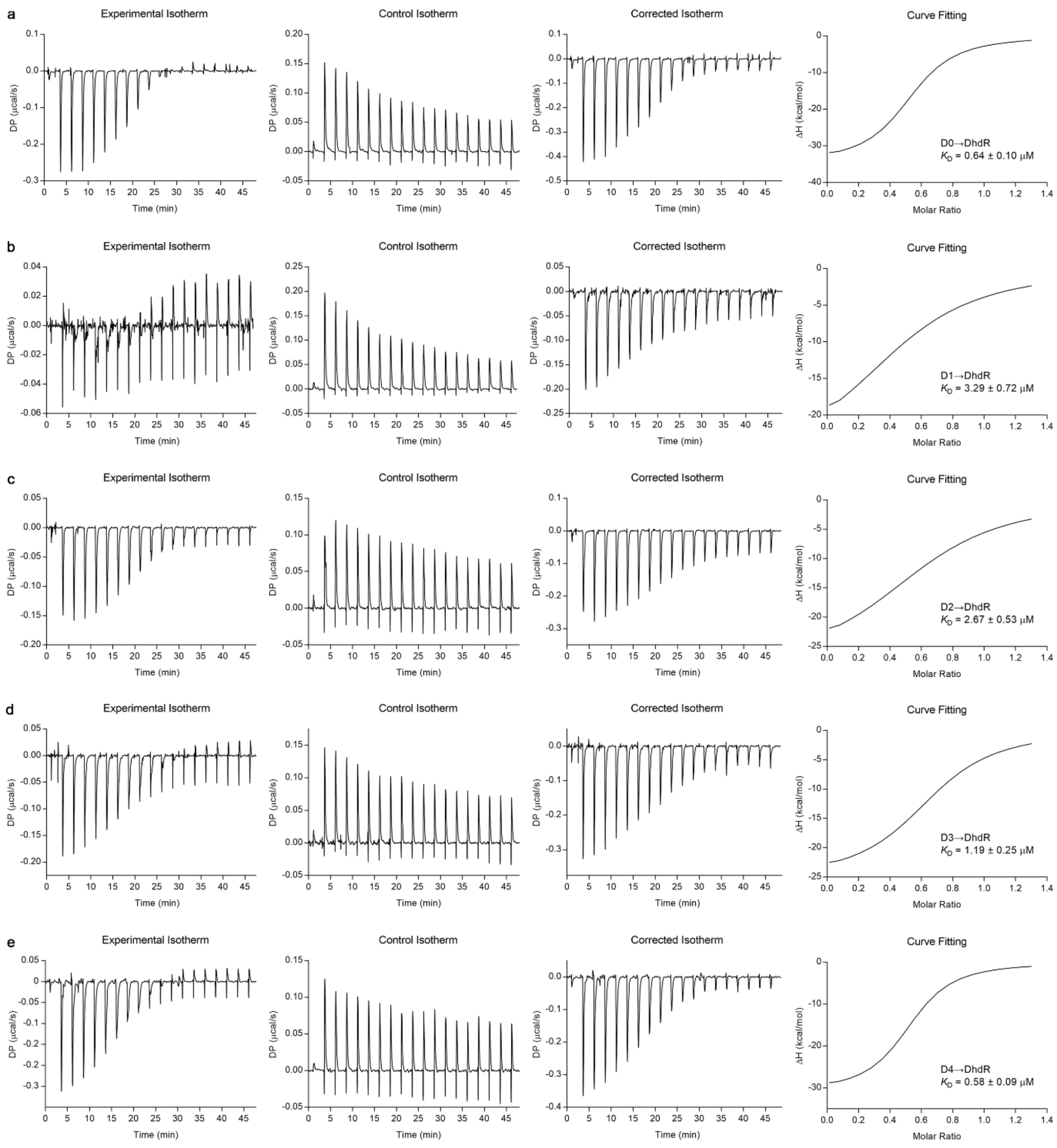

Supplementary Figure 13-1. Determination of affinities of DhdR with 27-bp DNA fragments containing DBS or DBS mutants by ITC. a, Titration of D0 into DhdR. **b**, Titration of D1 into DhdR. **c**, Titration of D2 into DhdR. **d**, Titration of D3 into DhdR. **e**, Titration of D4 into DhdR. 100 μM DNA fragment was titrated into 15 μM DhdR with 19 injections. The control experiment was performed by titrating the corresponding DNA fragment into reaction buffer. The net heat of the dilutions was corrected by subtracting the heat of the control point-to-point. A one site fitting model was used for curve fitting by using MicroCal PEAQ-ITC analysis software. Source data are provided as a Source Data file.

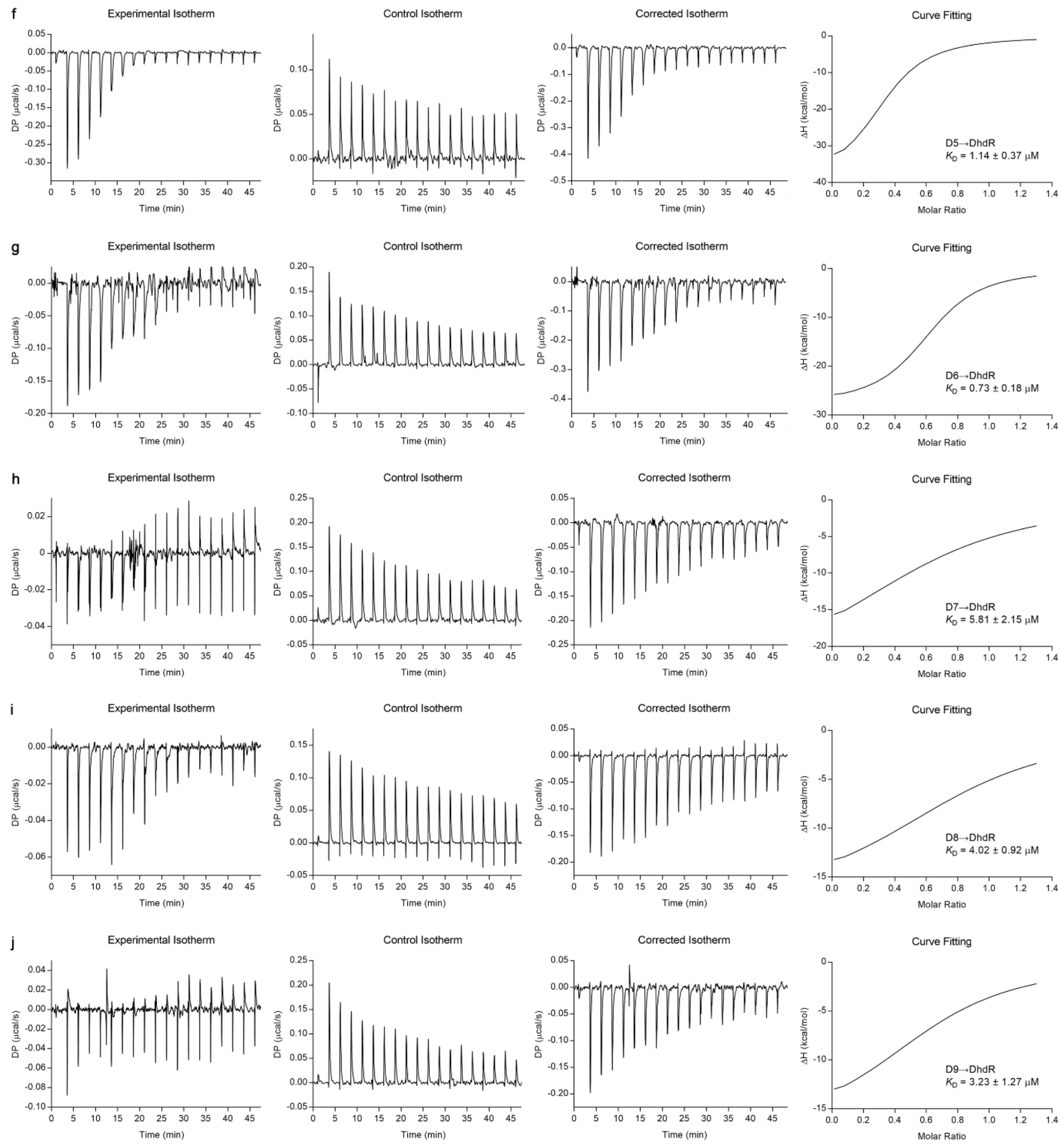

Supplementary Figure 13-2. Determination of affinities of DhdR with 27-bp DNA fragments containing DBS or DBS mutants by ITC. f, Titration of D5 into DhdR. **g**, Titration of D6 into DhdR. **h**, Titration of D7 into DhdR. **i**, Titration of D8 into DhdR. **j**, Titration of D9 into DhdR. 100 µM DNA fragment was titrated into 15 µM DhdR with 19 injections. The control experiment was performed by titrating the corresponding DNA fragment into reaction buffer. The net heat of the dilutions was corrected by subtracting the heat of the control point-to-point. A one site fitting model was used for curve fitting by using MicroCal PEAQ-ITC analysis software. Source data are provided as a Source Data file.

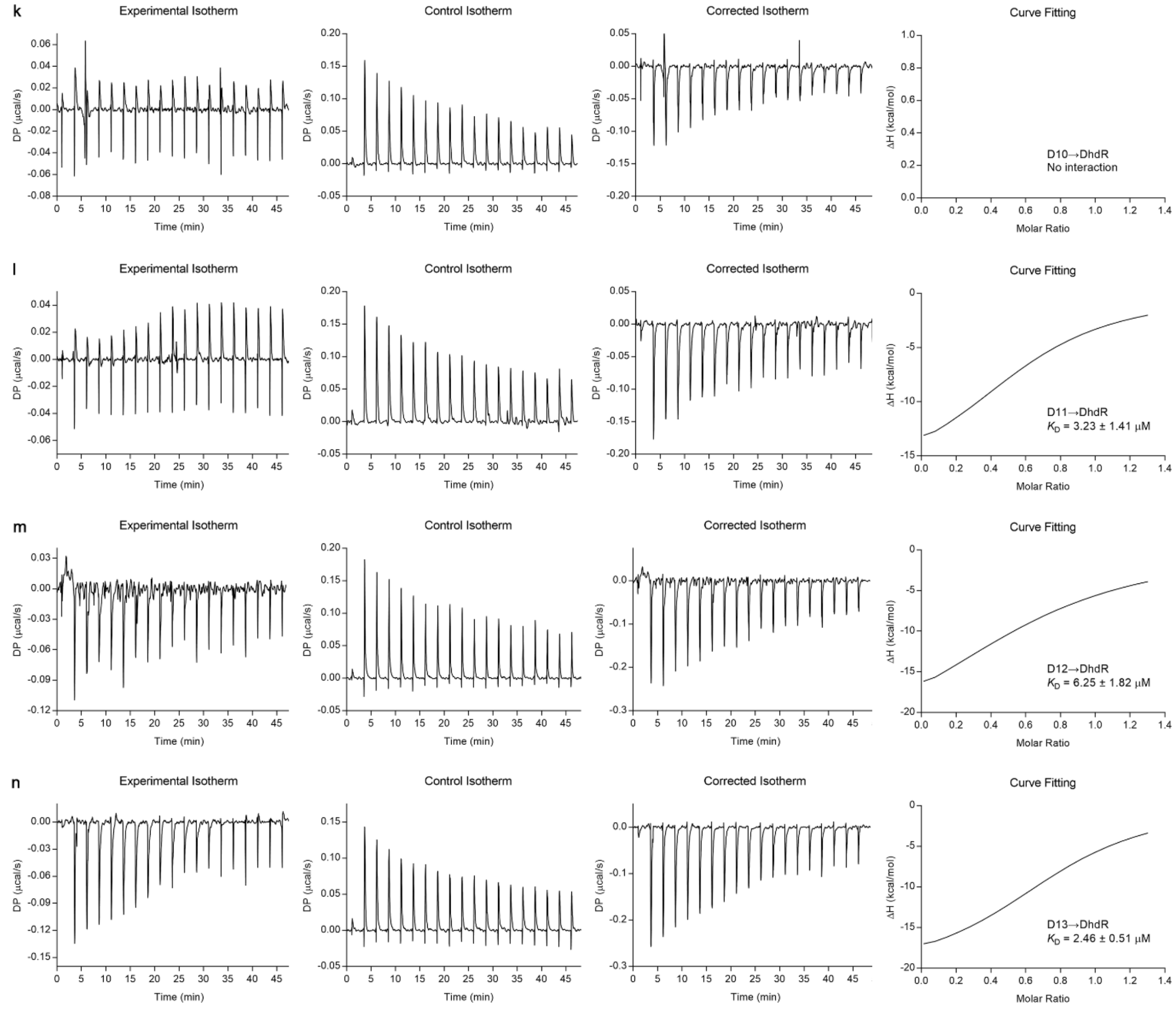

Supplementary Figure 13-3. Determination of affinities of DhdR with 27-bp DNA fragments containing DBS or DBS mutants by ITC. k, Titration of D10 into DhdR. **l**, Titration of D11 into DhdR. **m**, Titration of D12 into DhdR. **n**, Titration of D13 into DhdR. 100 μM DNA fragment was titrated into 15 μM DhdR with 19 injections. The control experiment was performed by titrating the corresponding DNA fragment into reaction buffer. The net heat of the dilutions was corrected by subtracting the heat of the control point-to-point. A one site fitting model was used for curve fitting by using MicroCal PEAQ-ITC analysis software. No apparent interaction was detected between DhdR and D10. Source data are provided as a Source Data file.

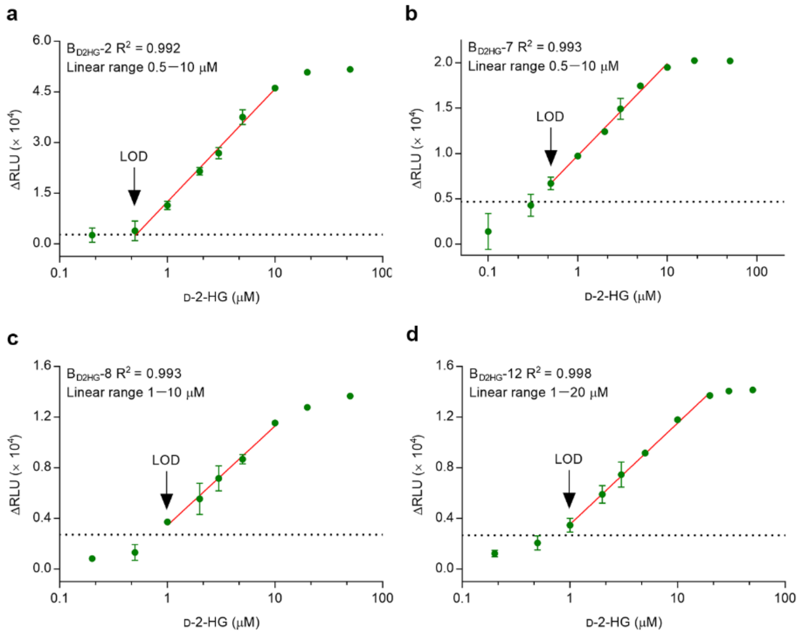

Supplementary Figure 14. The linear detection ranges of the different biosensors. a, B$_{D2HG}$-2; **b**, B$_{D2HG}$-7; **c**, B$_{D2HG}$-8; **d**, B$_{D2HG}$-12. Black dotted line is a reference line where the reduced luminescence signal (ΔRLU) is three times the background signal. Data shown are mean ± s.d. (n = 3 independent experiments). Source data are provided as a Source Data file.

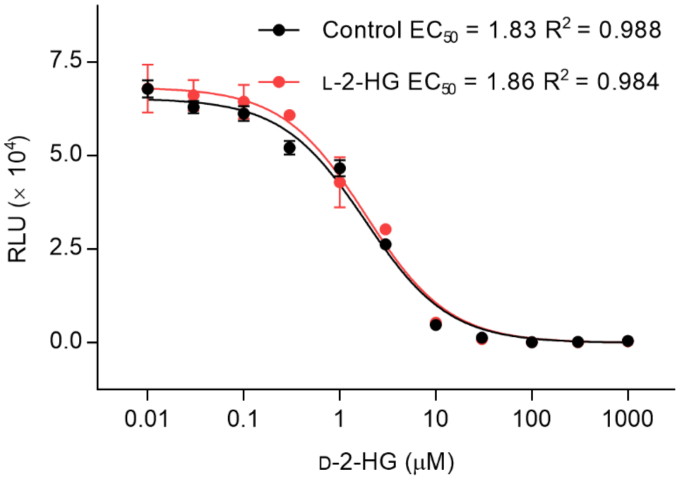

Supplementary Figure 15. Effects of L-2-HG in cell culture medium on the quantification of D-2-HG. The concentration of L-2-HG in cell culture medium was 1 mM. Dose-response curves of B_{D2HG}-1 were determined in 10-fold diluted cell culture medium in the presence (red line) or absence (black line) of L-2-HG. Data shown are mean ± s.d. (n = 3 independent experiments). Source data are provided as a Source Data file.

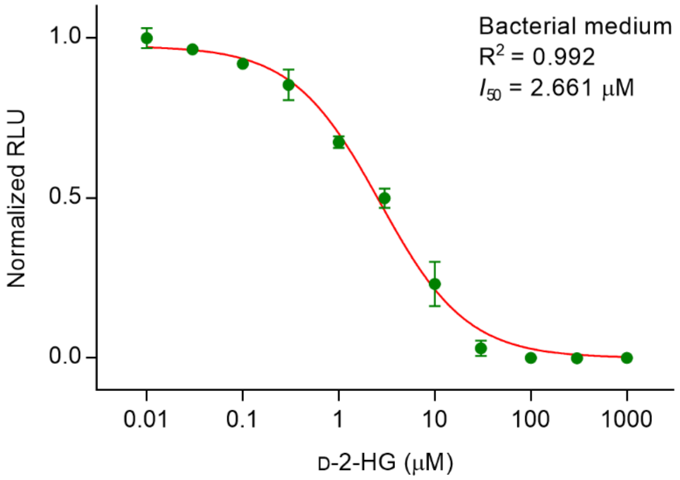

Supplementary Figure 16. Normalized dose-response curve of B_{D2HG}-1 in bacterial minimal medium. The corresponding luminescence signals are normalized to the maximal value. Data shown are mean ± s.d. (n = 3 independent experiments). Source data are provided as a Source Data file.

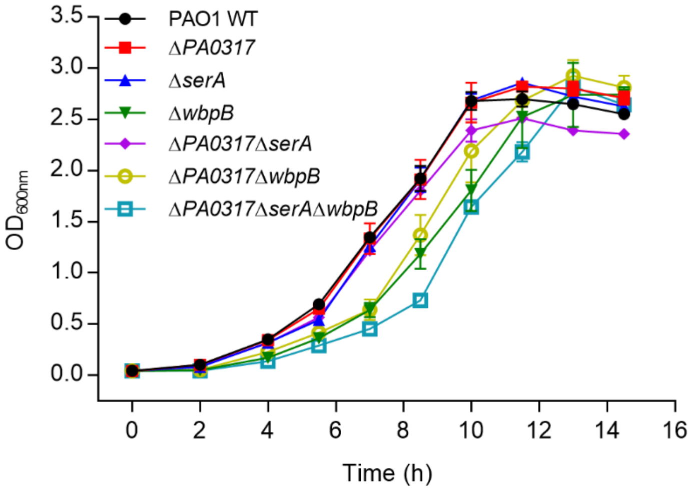

Supplementary Figure 17. Growth of *P. aeruginosa* PAO1 and its derivatives in minimal medium containing 3 g L^{-1} glucose and 4 mM L-serine. Data shown are mean ± s.d. (n = 3 independent experiments). Source data are provided as a Source Data file.

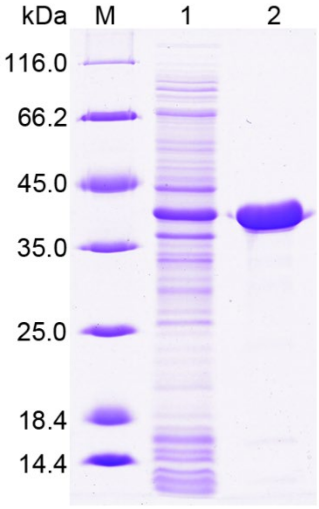

Supplementary Figure 18. SDS-PAGE analysis of purified *P. aeruginosa* WbpB. Lane M, molecular weight markers; lane 1, crude extract of *E. coli* BL21(DE3) harboring pETDuet-*wbpB*; lane 2, purified WbpB using a HisTrap column. Source data are provided as a Source Data file.

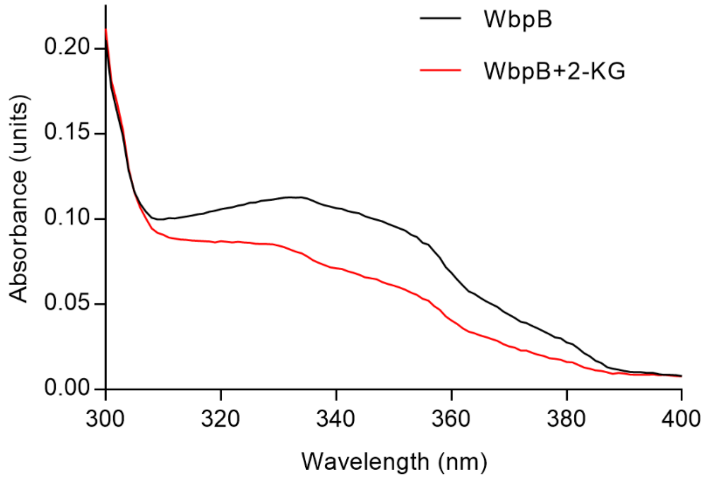

Supplementary Figure 19. Spectral analysis of WbpB. The UV-visible absorbance spectra of the purified WbpB was analyzed in 200 µL mixture containing 50 mM Tris-HCl buffer (100 mM NaCl, pH 7.4) and 0.4 mg WbpB before (black line) and after (red line) treated with 1.25 mM 2-KG for 2 h by using an EnSight Multimode Plate Reader (PerkinElmer, USA). Source data are provided as a Source Data file.

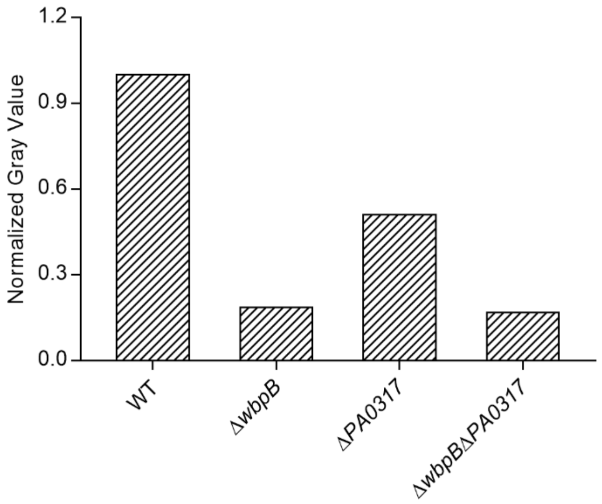

Supplementary Figure 20. Relative quantification of O-antigen polymers in LPS of *P. aeruginosa* PAO1 and its derivatives. The normalized gray values were obtained from the one representative experiment (**Fig. 6e**) of three independent experiments of silver-stained SDS-PAGE gel. The gray value analysis was performed by ImageJ 1.52p. Source data are provided as a Source Data file.

Supplementary Table 1 Occurrence of genomic context of *d2hgdh* in bacterial genomes.[a]

Organism	SerA	GntR	ETF	CdaR	LldP	Glycolate oxidase
**Alphaproteobacteria (341)**	**7**	**21**	**4**	**0**	**0**	**0**
Sinorhizobium meliloti AK83	−	+	−	−	−	−
Ochrobactrum anthropi ATCC 49188	−	+	−	−	−	−
Agrobacterium sp. H13-3	−	+	−	−	−	−
**Betaproteobacteria (441)**	**2**	**83**	**6**	**0**	**0**	**0**
Bordetella pertussis 137	−	+	−	−	−	−
Achromobacter denitrificans	−	+	−	−	−	−
Achromobacter xylosoxidans	−	+	−	−	−	−
**Deltaproteobacteria (63)**	**7**	**0**	**0**	**0**	**2**	**2**
Corallococcus coralloides DSM 2259	+	−	−	−	−	−
**Epsilonproteobacteria (159)**	**0**	**0**	**0**	**0**	**0**	**0**
**Gammaproteobacteria (351)**	**295**	**1**	**1**	**0**	**0**	**0**
Pseudomonas stutzeri A1501	+	−	−	−	−	−
Pseudomonas aeruginosa PAO1	+	−	−	−	−	−
Acinetobacter baumannii	+	−	−	−	−	−
**Actinobacteria (276)**	**1**	**4**	**9**	**0**	**1**	**2**
Pseudarthrobacter phenanthrenivorans Sphe3	−	+	−	−	+	−
**Bacilli (261)**	**0**	**14**	**0**	**70**	**9**	**142**
Bacillus amyloliquefaciens IT-45	−	−	−	−	−	+
Bacillus cereus NJ-W	−	−	−	+	−	+
Bacillus sp. X1(2014)	−	+	−	−	+	+
Paenibacillus sp. 32O-W	−	+	−	−	−	+
**Clostridia (88)**	**0**	**3**	**35**	**0**	**9**	**3**
Acetohalobium arabaticum DSM 5501	−	+	+	−	+	−
Carboxydothermus hydrogenoformans Z-2901	−	+	−	−	+	+
**Acidobacteria (1)**	**0**	**0**	**0**	**0**	**2**	**0**
**Bacteroidetes (35)**	**0**	**0**	**0**	**0**	**0**	**0**
**Ignavibacteriae (1)**	**0**	**0**	**0**	**0**	**0**	**1**
**Aquificae (16)**	**1**	**0**	**0**	**0**	**0**	**0**
**Candidate division NC10 (1)**	**0**	**0**	**0**	**0**	**0**	**0**
**Chloroflexi (6)**	**0**	**0**	**0**	**0**	**0**	**0**
**Cyanobacteria (20)**	**0**	**0**	**0**	**0**	**0**	**0**
**Deferribacteres (1)**	**0**	**0**	**0**	**0**	**0**	**0**
**Deinococcus-Thermus (19)**	**0**	**0**	**0**	**0**	**0**	**8**
**Erysipelotrichia (1)**	**0**	**0**	**0**	**0**	**0**	**0**
**Limnochordia (1)**	**0**	**0**	**0**	**0**	**0**	**1**
**Negativicutes (7)**	**0**	**0**	**6**	**0**	**0**	**0**
**Tissierellia (1)**	**0**	**0**	**1**	**0**	**0**	**0**
**Fusobacteriales (12)**	**0**	**0**	**9**	**0**	**1**	**0**
**Nitrospirae (1)**	**0**	**0**	**0**	**0**	**0**	**0**
**Acidithiobacillia (5)**	**0**	**0**	**0**	**0**	**0**	**0**
**Spirochaetes (16)**	**0**	**0**	**4**	**0**	**0**	**0**
**Synergistetes (2)**	**0**	**0**	**2**	**0**	**0**	**0**
**Tenericutes (2)**	**0**	**0**	**0**	**0**	**0**	**0**
**Thermobaculum (1)**	**0**	**0**	**0**	**0**	**0**	**0**
**Thermodesulfobacteria (4)**	**0**	**0**	**0**	**0**	**1**	**0**
**Thermotogae (6)**	**0**	**0**	**6**	**0**	**0**	**0**
**Verrucomicrobia (4)**	**1**	**0**	**0**	**0**	**0**	**0**
**Total: 2143**	**314**	**126**	**83**	**70**	**25**	**159**

[a]Representative species in several taxonomic groups of bacteria are shown as rows and the presence or absence of genes enconding the respective functional protein (columns) is shown by + or −. Numbers for taxonomic group rows indicate the number of species that have a gene ortholog.

Supplementary Table 2 Kinetic parameters of purified D2HGDH and SerA from *A. denitrificans* NBRC 15125[a].

Enzyme	Substrate	K_m (μM)	V_{max} (μM min^{-1})	k_{cat} (s^{-1})	k_{cat}/K_m (s^{-1} μM^{-1})
D2HGDH[b]	D-2-HG	31.16 ± 1.41	40,681.73 ± 946.03	6.90 ± 0.16	0.22 ± 0.00
SerA[c]	2-KG	57.88 ± 0.71	4,062.02 ± 41.74	5.79 ± 0.06	0.10 ± 0.00

[a]Data shown are mean ± s.d. (n = 3 independent experiments).

[b]The activity of D2HGDH was assayed at 30 ºC by determining the reduction of DCPIP spectrophotometrically at 600 nm in 800 μL reaction mixture containing 50 mM Tris-HCl buffer (pH 7.4), 0.01 mg mL^{-1} purified D2HGDH, 0.2 mM PMS, 0.05 mM DCPIP, and variable concentrations of D-2-HG.

[c]The activity of SerA was assayed at 30 ºC by measuring the oxidation of NADH spectrophotometrically at 340 nm in 800 μL reaction mixture containing 50 mM Tris-HCl buffer (pH 7.4), 0.05 mg mL^{-1} purified SerA, 0.2 mM NADH, and variable concentrations of 2-KG.

Supplementary Table 3 Assessment of the robustness of biosensor performance. Interference with biosensor function by a number of physiologically relevant compounds (at 100 μM).

Foreign substance[a]	Change of luminescence signal[b] (%)	Foreign substance	Change of luminescence signal (%)
K^+	2.94	Creatinine	0.15
NH_4^+	0.97	Sucrose	0.49
Na^+	0.06	Urea	1.31
Ca^{2+}	0.81	Ascorbic	1.17
Mg^{2+}	2.07	Leucine	1.99
Methionine	0.54	Valine	1.35
Alanine	0.51	Serine	0.72

[a]The concentration of inorganic substances or amino acids is 100 μM. The detecting solution (25 μL) contained 5 μL of each component: DhdR (0.3 nM), Bio-*dhdO* (1 nM), donor beads (20 μg mL^{-1}), acceptor beads (20 μg mL^{-1}) and various physiologically relevant compounds (100 μM).

[b]Data of change of luminescence signal shown are the mean value (n = 3 independent experiments).

Supplementary Table 4 Key parameters of the developed biosensors for D-2-HG assay in HBS-P buffer.

Parameter	B_{D2HG}-0	B_{D2HG}-1	B_{D2HG}-2	B_{D2HG}-7	B_{D2HG}-8	B_{D2HG}-12
K_D (μM)[a]	0.64	3.03	2.67	5.81	4.02	6.25
EC_{50} (μM)[b]	9.05	1.33	2.69	1.19	1.62	1.68
LOD (μM)[c]	0.50	0.10	0.50	1.00	1.00	1.00
Linear range (μM)	2−50	0.3−20	0.5−10	0.5−10	1−10	1−20

[a]The equilibrium dissociation constant (K_D) was determined by ITC and analyzed by using a single-site binding model.

[b]EC_{50} indicates the concentration of D-2-HG producing a 50% signal reduction.

[c]The limit of detection (LOD) was the minimal concentration of D-2-HG where the reduced luminescence signal (ΔRLU) is at least three times of the background signal[1].

Supplementary Table 5 Comparison of LC-MS/MS and B_{D2HG}-1 of D-2-HG detection.

Method	Cost ($/reaction)	Sample preparation	Availability of microplate	Samples/detection[c]	Ref.
LC-MS/MS	> 54.25[a]	Deproteinizaiton required	No	1	This study
B_{D2HG}-1	> 3.66[b]	No sample pre-treatment	Yes, 384-well plates	384	This study

[a]Calibrated based on the quoted price for sample testing from Core Facilities for Life and Environmental Sciences (State Key Laboratory of Microbial Technology, Shandong University). The cost of chiral derivatization prior to MS analysis is not included.

[b]Calibrated based on the purchase expense of AlphaScreen donor and acceptor beads and the quoted price for sample testing from Core Facilities for Life and Environmental Sciences (State Key Laboratory of Microbial Technology, Shandong University). The cost of biotinylated DNA and DhdR is not included.

[c]The maximum number of samples in a single detection.

Supplementary Table 6 Evaluation of the performance of B_{D2HG}-1 in quantification of D-2-HG in different biological samples.

Condition	Approach	Concentration (μM)[a]			Accuracy (%)[b]			Precision (RSD%)[c]
	Standard	150	1,500	3,500	150	1,500	3,500	
Serum	LC-MS/MS	142.21	1,596.44	3,629.37	94.81	106.43	103.70	5.98
	B_{D2HG}-1	135.42	1,494.89	3,737.51	106.13	104.62	101.43	2.31
Urine	LC-MS/MS	159.39	1,370.13	3,332.71	106.26	91.34	95.22	7.93
	B_{D2HG}-1	145.41	1,456.73	3,577.44	107.12	103.10	98.13	4.38
Cell medium	LC-MS/MS	173.59	1,777.85	4,512.84	115.73	118.52	128.94	5.75
	B_{D2HG}-1	150.49	1,378.03	3,740.92	99.42	99.63	102.10	1.49

[a]Concentration was the data detected from biological samples by B_{D2HG}-1. The standard concentrations were the spiked concentrations to biological sample.

[b]$\text{Accuarcy\%} = \frac{\text{Concentraion determined by LC-MS/MS or } B_{D2HG}\text{-1}}{\text{Standard concentration}}$.

[c]$\text{Precision\%} = \frac{\text{Standard derivation of accuracy}}{\text{Mean value of accuracy}}$.

Supplementary Reference

1. Li, S. *et al*. A platform for the development of novel biosensors by configuring allosteric transcription factor recognition with amplified luminescent proximity homogeneous assays. *Chem. Commun.* **53**, 99−102 (2017).
