# Supplementary material for: A d-2-hydroxyglutarate biosensor based on specific transcriptional regulator DhdR
Source: Nat Commun. 2021 Dec 7;12:7108. doi: 10.1038/s41467-021-27357-7 (PMC8651671; doi:10.1038/s41467-021-27357-7)
Supplement: Supplementary file 2 — Reporting Summary [file 41467_2021_27357_MOESM2_ESM.pdf]

nature portfolio

Corresponding author(s): Chao Gao and Ping Xu

Last updated by author(s): Nov 10, 2021

Reporting Summary

Nature Portfolio wishes to improve the reproducibility of the work that we publish. This form provides structure for consistency and transparency in reporting. For further information on Nature Portfolio policies, see our Editorial Policies and the Editorial Policy Checklist.

Statistics

For all statistical analyses, confirm that the following items are present in the figure legend, table legend, main text, or Methods section.

n/a	Confirmed	
☐	☒	The exact sample size (*n*) for each experimental group/condition, given as a discrete number and unit of measurement
☐	☒	A statement on whether measurements were taken from distinct samples or whether the same sample was measured repeatedly
☐	☒	The statistical test(s) used AND whether they are one- or two-sided<br>*Only common tests should be described solely by name; describe more complex techniques in the Methods section.*
☒	☐	A description of all covariates tested
☒	☐	A description of any assumptions or corrections, such as tests of normality and adjustment for multiple comparisons
☐	☒	A full description of the statistical parameters including central tendency (e.g. means) or other basic estimates (e.g. regression coefficient) AND variation (e.g. standard deviation) or associated estimates of uncertainty (e.g. confidence intervals)
☐	☒	For null hypothesis testing, the test statistic (e.g. *F*, *t*, *r*) with confidence intervals, effect sizes, degrees of freedom and *P* value noted<br>*Give P values as exact values whenever suitable.*
☒	☐	For Bayesian analysis, information on the choice of priors and Markov chain Monte Carlo settings
☒	☐	For hierarchical and complex designs, identification of the appropriate level for tests and full reporting of outcomes
☒	☐	Estimates of effect sizes (e.g. Cohen's *d*, Pearson's *r*), indicating how they were calculated

Our web collection on statistics for biologists contains articles on many of the points above.

Software and code

Policy information about availability of computer code

Data collection	The luminescence was measured by EnSight Multimode Plate Reader. Detailed data collection is described in the Methods.
Data analysis	Data analyses were conducted using Microsoft Excel 2016, Graphpad Prism 7 (Graphpad), and OriginPro 2016 (OriginLab). The distribution of D2HGDH in bacteria was checked by using BLAST 2.2.31. The isothermal titration calorimetry data were processed by using MicroCal PEAQ-ITC analysis software 1.1.0.1262. The imaging data were obtained by using CanoScan 9000F MarKII (Cano) and processed by using ImageJ 1.52p.

For manuscripts utilizing custom algorithms or software that are central to the research but not yet described in published literature, software must be made available to editors and reviewers. We strongly encourage code deposition in a community repository (e.g. GitHub). See the Nature Portfolio guidelines for submitting code & software for further information.

Data

Policy information about availability of data

All manuscripts must include a data availability statement. This statement should provide the following information, where applicable:

- Accession codes, unique identifiers, or web links for publicly available datasets
- A description of any restrictions on data availability
- For clinical datasets or third party data, please ensure that the statement adheres to our policy

The data generated in this study are provided in the Supplementary Information file and the Source Data file. Source data are provided with this paper.

Field-specific reporting

Please select the one below that is the best fit for your research. If you are not sure, read the appropriate sections before making your selection.

- [x] Life sciences
- [ ] Behavioural & social sciences
- [ ] Ecological, evolutionary & environmental sciences

For a reference copy of the document with all sections, see nature.com/documents/nr-reporting-summary-flat.pdf

Life sciences study design

All studies must disclose on these points even when the disclosure is negative.

Sample size	No sample size calculations were performed for this study. Sample sizes are indicated for each experiment and were chosen based on similar studies. Where statistical tests were applied, we selected n=3 as the minimum sample size.
Data exclusions	No data was excluded from the analysis.
Replication	All experiments were repeated on at least 3 separate samples using the reported methods. All attempts at replication were successful.
Randomization	The designed experiments in this study didn't include random experiments, and the experimental results were not randomized.
Blinding	Blinding is not relevant to this study: only one variable is tested in each experiment

Reporting for specific materials, systems and methods

We require information from authors about some types of materials, experimental systems and methods used in many studies. Here, indicate whether each material, system or method listed is relevant to your study. If you are not sure if a list item applies to your research, read the appropriate section before selecting a response.

Materials & experimental systems

n/a	Involved in the study	
x		Antibodies
	x	Eukaryotic cell lines
x		Palaeontology and archaeology
x		Animals and other organisms
x		Human research participants
x		Clinical data
x		Dual use research of concern

Methods

n/a	Involved in the study	
x		ChIP-seq
x		Flow cytometry
x		MRI-based neuroimaging

Eukaryotic cell lines

Policy information about cell lines

Cell line source(s)	The HEK293FT cell line and the HT1080 cell line was purchased from Procell Life Science&Technology Co., Ltd. The HEK293FT-IDH1/R132H cell line with stable overexpression of IDH1/R132H was constructed as described in this study.
Authentication	The HT1080 cell line was authenticated by Procell Life Science&Technology Co., Ltd. (Wuhan, China) using short tandem repeat (STR) markers. Authentication procedures were not used for the HEK293FT cell line and HEK293FT-IDH1/R132H cell line.
Mycoplasma contamination	Cells were not tested for mycoplasma contamination.
Commonly misidentified lines (See ICLAC register)	No commonly misidentified cell lines were used in the study. HEK293FT cells were employed as packaging cell line for lenti-virus preparation since these cells grow rapidly and express the packing proteins. In addition, HEK293FT cells are easy to handle, and can be readily infected by lenti-virus. Thus, the HEK293FT cells were used to construct the cell line with stable overexpression of IDH1/R132H.
