# Supplementary material for: A d-2-hydroxyglutarate biosensor based on specific transcriptional regulator DhdR
Source: Nat Commun. 2021 Dec 7;12:7108. doi: 10.1038/s41467-021-27357-7 (PMC8651671; doi:10.1038/s41467-021-27357-7)
Supplement: Supplementary file 3 — Description of Additional Supplementary Files [file 41467_2021_27357_MOESM3_ESM.pdf]

File name: Supplementary Data 1

Description: Strains and plasmids used in this study.

File name: Supplementary Data 2

Description: Oligonucleotides used in this study.
